# Suture-Related Fungal Interstitial Interface Keratitis in Deep Anterior Lamellar Keratoplasty: A Case Report

**DOI:** 10.7759/cureus.22508

**Published:** 2022-02-22

**Authors:** Premalatha Santhiran, Wan Haslina Wan Abdul Halim, Meng Hsien Yong

**Affiliations:** 1 Ophthalmology, Universiti Kebangsaan Malaysia Medical Centre, Kuala Lumpur, MYS

**Keywords:** suture-related keratitis, dalk, fungal keratitis, lamellar keratoplasty, interstitial interface keratitis

## Abstract

Interstitial interface keratitis (IIK) in lamellar keratoplasty is a term used to describe infectious keratitis that primarily involves the graft-host interface. It poses specific challenges due to impaired access for microbiological testing and poor penetration of antimicrobial drugs, as well as ease of deeper extension of the microorganism. A 33-year-old male with a medical history of left eye deep anterior lamellar keratoplasty (DALK) with keratoconus, subsequently complicated with steroid-induced glaucoma controlled with Xen tube insertion, presented with acute left eye pain and redness for two days due to one broken corneal graft suture at 5 o’clock position with infiltrate at the graft-host junction. He was treated for suture-related bacterial keratitis (culture-negative) with intensive single broad-spectrum topical antibiotic after suture removal. However, the condition worsened, with dense stromal infiltrate extending into the graft-host interface junction which further progressed to an endothelial plaque. Systemic and topical antifungal treatments were started with adjunctive intracameral and subconjunctival voriconazole before improvement was observed. The condition was resolved with localized scarring without the need for repeat keratoplasty. The best-corrected vision was maintained at 6/36 due to residual sutured-related astigmatism with no signs of corneal graft rejection. Lamellar keratoplasty poses an increased risk of fungal IIK even after several years if there is a predisposing factor e.g., steroid usage and broken suture. Timely diagnosis and intervention are the keys to ensure an optimal outcome.

## Introduction

Infective keratitis is one of the most severe complications which may occur post-corneal transplant, especially after penetrating keratoplasty. Long-term topical corticosteroid usage in keratoplasty and exposed or broken corneal sutures in corneal graft increase the risk of infection. Steroids also may mask the signs of infection for a long period of time and delay the diagnosis due to the slow growth of microorganisms [1].

In recent years, lamellar keratoplasty is getting more popular and is replacing penetrating keratoplasty. Deep anterior lamellar keratoplasty (DALK) is a relatively new surgical technique that selectively replaces the anterior portion of cornea down to the Descemet's membrane, preserving the Descemet's membrane and endothelial layer [2]. DALK has the advantages of avoiding most complications associated with ‘open sky’ or penetrating surgery, preserving the corneal endothelium, and reducing risk of graft rejections while maintaining some architectural integrity, as well as early and late corneal transplant complications [2,3].

In lamellar keratoplasty, e.g., DALK, there is a formation of an interface between the donor graft and recipient bed known as the graft-host interface. Infection that arises from this space in the deep stromal layer is known as interstitial interface keratitis (IIK) and poses a treatment challenge due to impaired access for microbiological testing and penetration of antimicrobial drugs [1,4]. Sharma et al. stated that IIK is a serious complication of lamellar keratoplasty which leads to severe visual loss, loss of globe integrity and may require penetrating keratoplasty as an end treatment [5]. In light of the adverse outcome and the differences in treatment protocol depending on the authors, this case report reviewed the management of IIK post-DALK as observed in our patient.

## Case presentation

A 33-year-old gentleman with no known medical illness underwent uncomplicated DALK in his left eye for keratoconus three years ago without complication. He was treated with tapering dosage of topical dexamethasone 0.1% for about one year which was then maintained at once daily frequency. However, the patient developed steroid-induced glaucoma and underwent minimally invasive glaucoma surgery with Xen tube insertion in the second year of DALK. Subsequently, no topical antiglaucoma was needed in later years.

**Figure 1:**
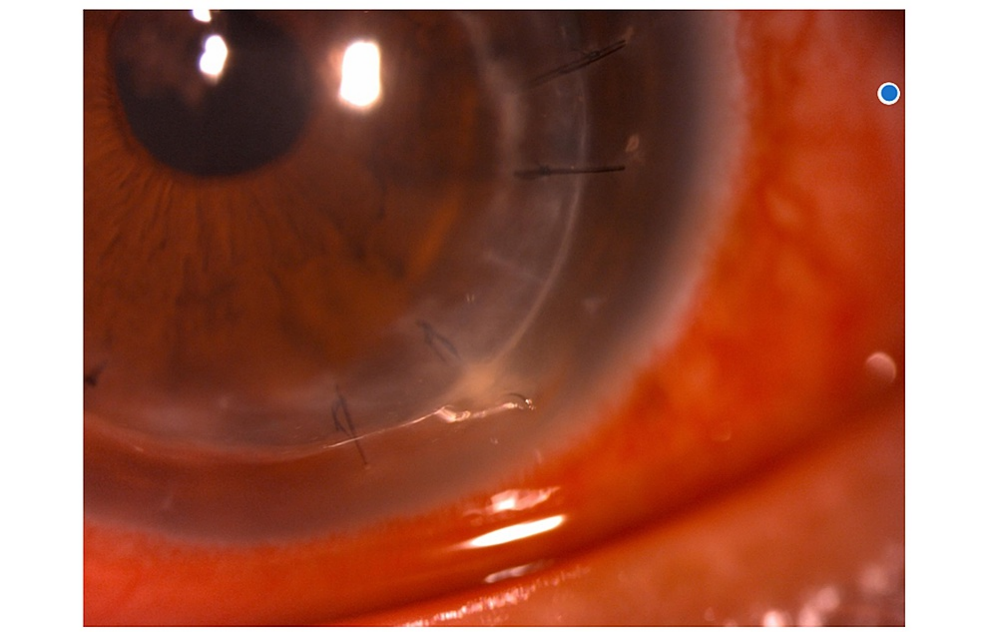
Broken suture with superficial infiltrate at graft-host junction.

He presented to the emergency eye clinic three years after the DALK with acute onset painful left red-eye for two days. Slit-lamp biomicroscopic examination revealed a broken corneal graft suture at 5 o’clock position with white to cream-colored deposit (infiltrate) at suture area over the superficial graft-host junction with clear graft (Figure 1). Intraocular pressure ranged above 30 mmHg. Otherwise, there were no epithelial defects nor evidence of wound leak, and the anterior chamber was quiet without hypopyon or signs of endophthalmitis.

**Figure 2:**
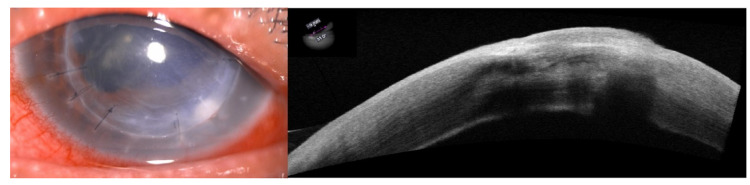
Stromal infiltrate was extending to the interface space from the initial graft-host junction, with intact uninvolved epithelium.

He was treated for suture-related bacterial keratitis with suture removal and then followed by monotherapy broad-spectrum topical antibiotic (gutt. moxifloxacin 0.5%) two hourly with two topical antiglaucoma medications and continued on low dose topical steroid (gutt. loteprednol 0.5%) once daily on the left eye (Figure 2). However, the eye condition did not improve after about one week of treatment and stromal infiltrate was seen extending to the interface space from the initial graft-host junction, with intact uninvolved epithelium. Thus, a corneal scraping was taken (culture-negative) and the patient was started with intensive dual topical antibiotics (gutt. gentamicin 0.9% and gutt. cefuroxime 5%) hourly around the clock, oral non-steroidal antiinflammatory drug (NSAID) (ibuprofen 400 mg TDS) for inflammation, oral doxycycline 100 mg OD, and oral ascorbic acid 1 g OD.

**Figure 3:**
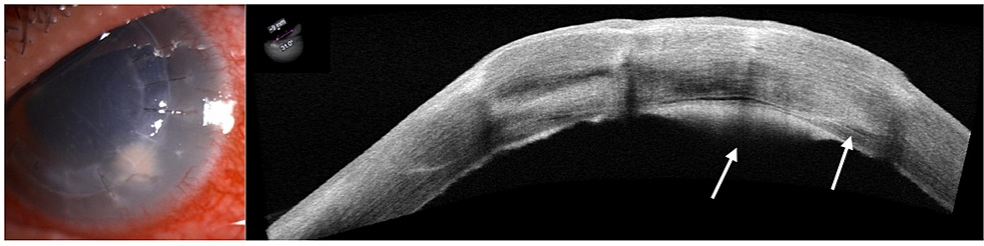
Endothelial plaque with dense infiltrate involving graft-host interface (arrow), with intact epithelium.

Despite this intensive antibacterial treatment, and the initial corneal tissue culture and sensitivity showing no growth, the clinical findings worsened with denser stromal infiltrate and formation of endothelial plaque (Figure 3). The corneal scraping was repeated and aqueous fluid was taken under the slit-lamp, but the culture again showed no growth or fungal staining. As the condition worsened, topical amphotericin B 0.5% and voriconazole 2% were started. Intracameral voriconazole 0.05% and subconjunctival voriconazole 2% were administered twice as an adjunctive. Oral voriconazole was initially started with 400 mg BD for a day followed by 200 g BD for a week. As the patient developed deranged liver function test with raised alanine aminotransferase (ALT), oral antifungal had to be discontinued.

**Figure 4:**
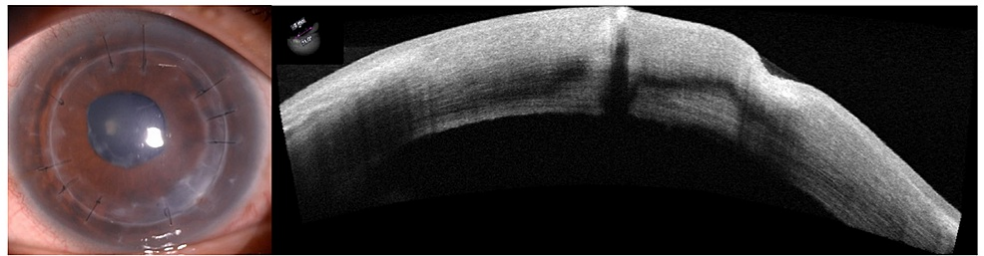
After six weeks of treatment, the infiltrate resolved with scarring and graft thinning.

After a few days of antifungal therapy, cyclosporine topical immunosuppressant ointment 0.1% was added as slow improvement was observed. Meanwhile, the topical antifungal and antibiotics were tapered slowly. After about six weeks of treatment, the infiltrates and endothelial plaque contracted and scarred, with minimal graft thinning and vascularization (Figure 4). The antifungal, antibiotic, antiglaucoma, and immunosuppressant were all tapered and stopped accordingly and changed back to maintenance once daily steroid eyedrop with artificial tears. The vision was maintained at 6/36 with residual suture-related astigmatism. Otherwise, there were no signs of graft rejection or recurrence of infection seen one year after completion of the treatment.

## Discussion

Interstitial interface keratitis (IIK) between the donor and host cornea following lamellar keratoplasty is a rare complication. Sharma et al. found that microbial keratitis developed in post-keratoplasty in 15 cases out of 135, but only three involved the interface area [6]. The most common causative pathogen was fungal (62%) dominated by Candida sp (45%) followed by Gram-positive, Gram-negative, and herpes simplex virus [1,7]. The Eye Bank Association of America reported an accumulated frequency of post-keratoplasty (all types) infection of 0.0026% for fungal and bacterial agents together, with a higher rate of fungal isolates (63%). The frequency of fungal infections after lamellar keratoplasty was nearly double that of penetrating keratoplasty (PK), and the rate of fungal infections after DALK was 0.052% and 0.022% after Descemet's stripping endothelial keratoplasty (DSAEK) [4]. Gao et al. suggested that tissue manipulation and corneal graft culture medium act as a contributing factor whereas some studies said that the tissue manipulation either in the eye bank or in the operating room does not seem to influence the post-operative risk of bacterial or fungal infection as seen in our case where the infection only happened three years after the DALK surgery, which was not related to tissue manipulation [1,4].

The time from surgery to presentation varies. Interface keratitis can present even from day one and up to several months post-uncomplicated keratoplasty. Late-onset IIK following lamellar keratoplasty is unlikely caused by Candida species unless there is a history of trauma or intervention [8]. The risk factors for IIK include loose sutures, topical corticosteroid, contaminated donor corneal tissue, chronic epithelial defect whereas the differential diagnosis includes graft rejection, epithelial ingrowth, and interface deposits [1,9]. Our case is late-onset IIK, as the infection only developed three years post-DALK, predisposed with suture abscess with loose suture. Slit-lamp examination of fungal keratitis is usually evidenced by intrastromal opacity with mild inflammation in the early stage [1]. Even though the cultures of the corneal scraping in our case were negative on two occasions, the clinical signs and treatment response were strongly suggestive of fungal keratitis.

Microbiological and histopathological examinations are the mainstay of investigation for diagnosis of IIK followed by anterior segment-optical coherence tomography (AS-OCT) and confocal microscopy to help for the confirmation and to monitor the disease progress [10]. Sharma et al. stated that interface keratitis can be in deep-seated locations and may not be readily available for corneal scraping [10]. This can be explained why the culture was negative in our patient despite the signs suggestive of fungal features. Moreover, the infections can be masked by long-term concomitant steroid and antibiotic usage pos-operatively. At the same time, aqueous sampling in establishing microbiological diagnosis is not so conclusive as shown in this case. Fontana et al. observed that there was no growth in aqueous culture in post-DALK interface keratitis [11]. AS-OCT is a non-invasive tool that can be used to determine the location, extend of infiltrates, and to monitor the progression, with potential advantage in differentiating between bacterial and fungal keratitis [10]. Soliman et al. stated that AS-OCT shows the presence of localized small stromal cystic spaces in localized stromal necrosis and full-thickness large stromal cystic spaces in diffuse stromal necrosis in fungal keratitis [12-14].

Treatment options for IIK can be divided into medical and surgical therapies. At initial presentation, empirical broad-spectrum antimicrobial therapy must be started for sterilization phase. In cases of suspected fungal keratitis, both systemic and topical antifungals such as natamycin, fluconazole, amphotericin B, and voriconazole can be considered during initial presentation itself. The medical therapy could be changed according to clinical response and microbiological findings on its growth and sensitivity. Steroid therapy has always been controversial in infectious keratitis and generally contraindicated in fungal infection [15,16]. Other non-steroidal immunomodulators should be considered in fungal IIK for the benefit of graft protection from inflammatory damage or subsequent rejection [17].

However, the deep location of infiltrates in the interface challenges the penetration of eye drops. Medical therapy alone is typically not very effective, due to the deeper location of the infection. Gao et al. reported that 63% of IIK failed medical therapy and required surgical therapy [1]. Thus, surgical intervention is often considered especially in fungal keratitis [10]. Options of surgical therapy include interface scraping, antibiotic irrigation, intracameral or intrastromal injection, interface injection, lamellar graft exchange, and therapeutic penetrating keratoplasty (TPK) [13]. Intrastromal and intracameral injections depend on the location of the infiltrates. Although complications can occur post-injection, the lesion can potentially heal without surgical intervention. Tu and Hou reported central detachment of the donor lenticule in post-DSAEK fungal interface keratitis with peripheral attachment, whereas Araki-Sasaki et al. reported that interface fluid injection resulted in detachment of the donor lenticule from the stroma and adversely affected graft survival. Despite the graft detachment, the infection healed with stromal opacity in both cases [14,18]. Nahum et al. stated that intracameral injections of ceftazidime, amikacin, vancomycin, and voriconazole in appropriate concentrations in five cases did not lead to any curative effect, while Kitzmann et al. reported a small perforation post-intracameral injections of amphotericin B [19,20]. Therapeutic penetrating keratoplasty is viewed as the best choice by some authors for speedy visual rehabilitation and graft survival, as the infection is deep-seated with high recurrence risk despite adequate medical treatment [10,13].

In our case, the multi-route combination of topical, subconjunctival, and intracameral antifungals, together with the initial topical prophylactic antibacterial, successfully treated the infection without the need for penetrating keratoplasty. Timely initiation of immunomodulator further protected the graft from rejection.

## Conclusions

Eyes with a history of lamellar keratoplasty have the risk of fungal IIK even after years of surgery if there is presence of predisposing factor. Timely diagnosis and intervention are the keys to ensure optimal outcomes in IIK without the need for repeating keratoplasty. AS-OCT is a rapid non-invasive test that can provide high-resolution images of graft-host interface junction at all times. The early initiation of antifungal therapy is beneficial and highly recommended even though the culture is not suggestive. It plays a major role in the outcome of the surgery. In the future, it is beneficial to send the suture to involve for microbiological analysis in suture-related keratitis patients.

## References

[1] Gao Y, Li C, Bu P, Zhang L, Bouchard CS (2019). Infectious interface keratitis (IIK) following lamellar keratoplasty: a literature review. Ocul Surf.

[2] Sarnicola V, Toro P, Gentile D, Hannush SB (2010). Descemetic DALK and predescemetic DALK: outcomes in 236 cases of keratoconus. Cornea.

[3] Liu H, Chen Y, Wang P, Li B, Wang W, Su Y, Sheng M (2015). Efficacy and safety of deep anterior lamellar keratoplasty vs. penetrating keratoplasty for keratoconus: a meta-analysis. PLoS One.

[4] Fontana L, Moramarco A, Mandarà E, Russello G, Iovieno A (2019). Interface infectious keratitis after anterior and posterior lamellar keratoplasty. Clinical features and treatment strategies. A review. Br J Ophthalmol.

[5] Sharma N, Agarwal PC, Kumar CS, Mannan R, Titiyal JS (2011). Microbial keratitis after descemet stripping automated endothelial keratoplasty. Eye Contact Lens.

[6] Sharma N, Gupta V, Vanathi M, Agarwal T, Vajpayee RB, Satpathy G (2004). Microbial keratitis following lamellar keratoplasty. Cornea.

[7] Lyall DA, Srinivasan S, Roberts F (2012). A case of interface keratitis following anterior lamellar keratoplasty. Surv Ophthalmol.

[8] Kodavoor SK, Dandapani R, Kaushik AR (2016). Interface infectious keratitis following deep anterior lamellar keratoplasty. Indian J Ophthalmol.

[9] Kanavi MR, Foroutan AR, Kamel MR, Afsar N, Javadi MA (2007). Candida interface keratitis after deep anterior lamellar keratoplasty: clinical, microbiologic, histopathologic, and confocal microscopic reports. Cornea.

[10] Sharma N, Kaur M, Titiyal JS, Aldave A (2021). Infectious keratitis after lamellar keratoplasty. Surv Ophthalmol.

[11] Fontana L, Parente G, Pede BD, Tassinari G (2007). Candida albicans interface infection after deep anterior lamellar keratoplasty. Cornea.

[12] Soliman W, Fathalla AM, El-Sebaity DM, Al-Hussaini AK (2013). Spectral domain anterior segment optical coherence tomography in microbial keratitis. Graefes Arch Clin Exp Ophthalmol.

[13] Murthy SI, Jain R, Swarup R, Sangwan VS (2013). Recurrent non-tuberculous mycobacterial keratitis after deep anterior lamellar keratoplasty for keratoconus. BMJ Case Rep.

[14] Tu EY, Hou J (2014). Intrastromal antifungal injection with secondary lamellar interface infusion for late-onset infectious keratitis after DSAEK. Cornea.

[15] Palioura S, Henry CR, Amescua G, Alfonso EC (2016). Role of steroids in the treatment of bacterial keratitis. Clin Ophthalmol.

[16] Knutsson KA, Iovieno A, Matuska S, Fontana L, Rama P (2021). Topical corticosteroids and fungal keratitis: a review of the literature and case series. J Clin Med.

[17] Lyle WA, Jin GJ (1999). Interface fluid associated with diffuse lamellar keratitis and epithelial ingrowth after laser in situ keratomileusis. J Cataract Refract Surg.

[18] Araki-Sasaki K, Fukumoto A, Osakabe Y, Kimura H, Kuroda S (2014). The clinical characteristics of fungal keratitis in eyes after Descemet's stripping and automated endothelial keratoplasty. Clin Ophthalmol.

[19] Nahum Y, Russo C, Madi S, Busin M (2014). Interface infection after descemet stripping automated endothelial keratoplasty: outcomes of therapeutic keratoplasty. Cornea.

[20] Kitzmann AS, Wagoner MD, Syed NA, Goins KM (2009). Donor-related Candida keratitis after Descemet stripping automated endothelial keratoplasty. Cornea.

